# Provitamin A Biofortification of Durum Wheat through a TILLING Approach

**DOI:** 10.3390/ijms20225703

**Published:** 2019-11-14

**Authors:** Francesco Sestili, Maria Dolores Garcia-Molina, Gianluca Gambacorta, Romina Beleggia, Ermelinda Botticella, Pasquale De Vita, Daniel Valentin Savatin, Stefania Masci, Domenico Lafiandra

**Affiliations:** 1 Department of Agriculture and Forest Sciences, University of Tuscia, 01100 Viterbo, Italy; francescosestili@unitus.it (F.S.); lolagmolina@gmail.com (M.D.G.-M.); gianluca.ga89@gmail.com (G.G.); e.botticella@unitus.it (E.B.); daniel.savatin@unitus.it (D.V.S.); masci@unitus.it (S.M.); 2 Council for Agricultural Research and Economics (CREA), Research Centre for Cereal and Industrial Crops (CREA-CI), 71122 Foggia, Italy; romina.beleggia@crea.gov.it (R.B.); pasquale.devita@crea.gov.it (P.D.V.)

**Keywords:** durum wheat, β-carotene, TILLING, biofortification, vitamin A deficiency

## Abstract

Macro- and micronutrients, essential for the maintenance of human metabolism, are assimilated daily through the diet. Wheat and other major cereals are a good source of nutrients, such as carbohydrates and proteins, but cannot supply a sufficient amount of essential micronutrients, including provitamin A. As vitamin A deficiency (VAD) leads to several serious diseases throughout the world, the biofortification of a major staple crop, such as wheat, represents an effective way to preserve human health in developing countries. In the present work, a key enzyme involved in the branch of carotenoids pathway producing β-carotene, lycopene epsilon cyclase, has been targeted by a Targeting Induced Local Lesions in Genomes (TILLING) approach in a “block strategy” perspective. The null mutant genotype showed a strong reduction in the expression of the *lcyE* gene and also interesting pleiotropic effects on an enzyme (β-ring hydroxylase) acting downstream in the pathway. Biochemical profiling of carotenoids in the wheat mutant lines showed an increase of roughly 75% in β-carotene in the grains of the complete mutant line compared with the control. In conclusion, we describe here the production and characterization of a new wheat line biofortified with provitamin A obtained through a nontransgenic approach, which also sheds new light on the molecular mechanism governing carotenoid biosynthesis in durum wheat.

## 1. Introduction

Vitamins and minerals are essential elements for growth and metabolism and they are referred to as micronutrients, since they are necessary in small doses. The World Health Organization (WHO) has recently estimated that more than 2 billion people are suffering from vitamin and mineral deficiencies, in particular, vitamin A, iodine (I), iron (Fe), and zinc (Zn) [1]. Typical diets of low- and middle-income countries are based on staple crops, such as rice (*Oryza sativa*), corn (*Zea mays*), wheat (*Triticum*), potato (*Solanum tuberosum*), and soy (*Glycine max*). Although these crops can satisfy the daily caloric requirement, they do not provide the right quantities of essential nutrients involved in the maintenance and proper functioning of metabolism that is required for a good state of health [2].

Vitamin A deficiency (VAD) has been defined by the WHO as the major nutritional issue afflicting middle- and low-income countries. This deficiency is caused by inadequate chronic dietary intake of vitamin A and its metabolic precursors, such as β-carotene. Vitamin A (retinol) and its derivatives are key nutrients required for the proper functioning of the visual, immune, and reproductive systems; the maintenance of cell function and differentiation; the maintenance of epithelial integrity; and the process of hematopoiesis [3]. Insufficient chronic intake of vitamin A is the main cause of VAD, especially in the physiological periods in which the need for this nutrient is high, for example, during childhood, pregnancy, and lactation [4]. VAD disorders range from ocular manifestations of xerophthalmia to generic disorders, such as night blindness; immune system malfunction; anemia; and increased infant mortality caused by measles, diarrhea, and general infections [5,6]. Since vitamin A cannot be synthesized by humans, this micronutrient or its metabolic precursors must be provided by the diet [7].

Many of the currently available staple crops are characterized by low amounts of carotenoids, which are not sufficient to meet human needs and vary between 3 and 6 mg/day [8]. For this reason, numerous studies have focused on vitamin A biofortification of staple crops since 1990s. In particular, the genetic manipulation of carotenoid metabolism, aimed at increasing the accumulation of provitamin A within the kernels of major cereals, such as corn, rice, and wheat, can be a powerful tool to reduce VAD, especially for those populations which base their diet on one or a few crops [9].

In higher plants, all carotenoids derive from the plastidial metabolic pathway of methyl-erythrose 4-phosphate (MEP), in which glyceraldehyde-3-phosphate and pyruvate act as the initial substrates for the formation of geranylgeranyl pyrophosphate (GGPP), the common precursor for the biosynthesis of carotenoids and numerous terpenoid (or isoprenoid) compounds [10]. The first committed step of carotenoid synthesis is the condensation of two GGPP molecules by the phytoene synthase (PSY) with the formation of 15-*cis*-phytoene. Phytoene is converted into lycopene by two desaturation reactions catalyzed by phytoene desaturase (PDS) and ζ-carotene desaturase (ZDS) enzymes. These enzymes give rise to poly-*cis* compounds which are converted to all-*trans*-lycopene by the enzymes ζ-carotene isomerase (ZISO) and carotene isomerase (CRTISO) or by photoisomerization [8]. Lycopene constitutes the branching point of the MEP pathway, as it acts as a substrate for two different cyclases: lycopene ε-cyclase (LCYE) and lycopene β-cyclase (LCYB). α-carotene is produced when the two enzymes (LCYE and LCYB) act together at the two ends of the lycopene molecule adding two rings (β, ε branch). β-carotene is formed when LCYB acts alone to form a bicyclic molecule (β, β branch). α- and β-carotene are subsequently hydroxylated to produce lutein and zeaxanthin, respectively. Hydroxylation reactions are performed by β-ring hydroxylases (HYD) and heme-containing cytochrome P450 carotene ε-ring carotene hydroxylase (CYP) [8]. *Trans* (E)-lutein is the most abundant carotenoid in durum wheat kernels, while zeaxanthin, α-carotene, β-cryptoxanthin, and β-carotene are present only in trace amounts.

Since 1990, numerous works, focused on the metabolic engineering of carotenoid pathways in crop plants, have been published. The different strategies applied were reviewed by Giuliano et al. [11,12] and can be summarized as follows:

(1) “Push” strategies, acting on the metabolic upstream flow of the biosynthetic pathway, through the overexpression of one or more enzymes involved in the initial reactions. These strategies have greater efficiency and they have allowed for an increase in the content of carotenoids from 100 to 1000 times higher than in wild-type crops [13]. The best examples of this approach are the golden crops (canola, rice, potatoes, maize, cassava, wheat, and sorghum) with a high content of β-carotene [11].

(2) “Block” strategies, in which the accumulation of the metabolite of interest is obtained by silencing of the genes located downstream along the metabolic pathway or of those involved in competitive pathways. This approach gave rise to several biofortified crops, including potato cultivars with a high content of β-carotene and zeaxanthin [14].

The push and block strategies can be combined in a single event, in which the overexpression of phytoene synthase combined with the silencing of carotene hydroxylase allows for obtaining a high increase of β-carotene in transgenic wheat lines [15].

(3) Strategies aimed at increasing the number and size of cellular compartments responsible for carotenoid accumulation. For example, manipulation of the light perception/transduction pathways or the abscisic acid pathway caused an increase in the number and size of plastids in tomato, with a consequent greater accumulation of lycopene in berries [16]. In general, these strategies have produced a twofold increase in the total content of carotenoids [12].

(4) Strategies aimed at increasing postharvest carotenoid stability. In cereal kernels, carotenoids are subject to degradation by enzymes such as oxygenases and lipoxygenases. The deletion of the loci encoding lipoxygenases increased the stability of carotenoids during storage and processing [17,18]. This approach can be used to further improve the content and stability of carotenoids present in crops obtained through push and block strategies.

The aim of this work was to increase the bioavailability of β-carotene (precursor of vitamin A) in durum wheat kernels through a nontransgenic “block approach”. In particular, the lycopene substrate was directed towards the β-carotene synthesis pathway by targeting the genes coding LCYE enzymes. A Targeting Induced Local Lesions in Genomes (TILLING) strategy [19] was used to introduce knockout mutations on *lcyE* genes. Deleterious mutations were identified in an in silico TILLING population [20] derived from the durum wheat cultivar Kronos.

## 2. Results

### 2.1. Comparison of LCYE Amino Acid Sequences between Wheat and Other Species

**Figure 1:**
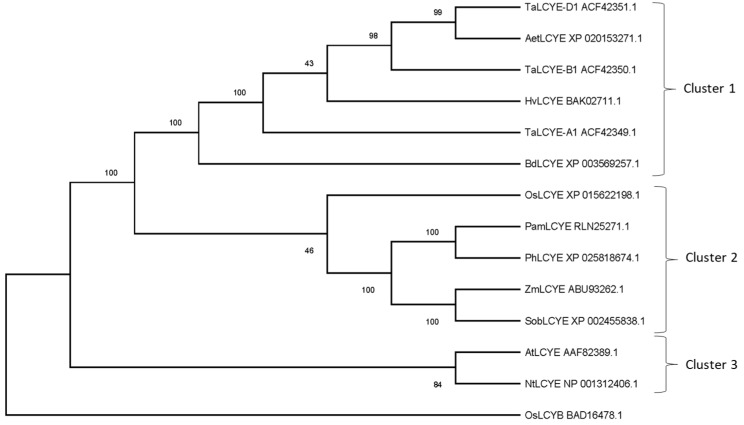
Phylogenetic analysis of the lycopene ε-cyclase (LCYE) protein of different plant species. Bootstrap values relating to each node are shown. Ta: *Triticum aestivum* (GenBank accessions ACF42349.1, ACF42350.1, and ACF42351.1); Aet: *Aegilops tauschii* (GenBank accession XP_020153271.1); Hv: *Hordeum vulgare* (GenBank accession BAK02711.1); Bd: *Brachypodium distachyon* (GenBank accession XP_003569257.1); Os: *Oryza sativa* (GenBank accessions XP_015622198.1 and BAD16478.1); Pam: *Panicum miliaceum* (GenBank accession RLN25271.1); Ph: *Panicum halli* (GenBank accession XP_02581867.1); Zm: *Zea mays* (GenBank accession ABU93262.1); Sob: *Sorghum bicolor* (GenBank accessionXP_002455838.1); At: *Arabidopsis thaliana* (GenBank accession AAF82389); and Nt: *Nicotiana tabacum* (NP_001312406.1).

The classification based on Gene Ontology showed that all the analyzed sequences of *lcyE* genes had the same molecular function involved in the biosynthetic pathway of carotenoids (GO: 0016117). In addition, all the selected sequences contained two important conserved domains: the TIGR01790 domain, member of the protein superfamily cl27555 (carotene-cycl superfamily), and the PLN02697 domain belonging to the superfamily cl21454 (NADB_Rossmann superfamily). The TIGR01790 domain is typical of the lycopene beta and epsilon cyclase enzymes, while the NADB domain has also been found in numerous dehydrogenases involved in metabolic pathways such as glycolysis and in several redox enzymes. This domain (V/IXGXGXXGXXXA), involved in the interaction with the NAD/FAD cofactors (Armstrong and Hearst, 1996), was included in the dinucleotide binding region and, together with the adjacent residues, was well preserved in all analyzed sequences (Figure S1). In order to evaluate the evolutionary history of LCYE enzymes in different plant species, a phylogenetic analysis was carried out using the MEGAX software (version 10.0.5) (Figure 1). In the phylogenetic tree, it was possible to distinguish three different clusters. The first included the species *Triticum aestivum*, *Aegilops tauschii*, *Hordeum vulgare*, and *Brachypodium distachyon*. The second cluster included the species *Panicum miliaceum*, *Panicum halli*, *Zea mays*, *Sorghum bicolor*, and *Oryza sativa*. The third cluster was represented by the model organisms *Arabidopsis thaliana* and *Nicotiana tabacum*.

### 2.2. Identification of TILLING Durum Wheat Lines with Knockout Mutation on the lcyE Homeoalleles

**Figure 2:**
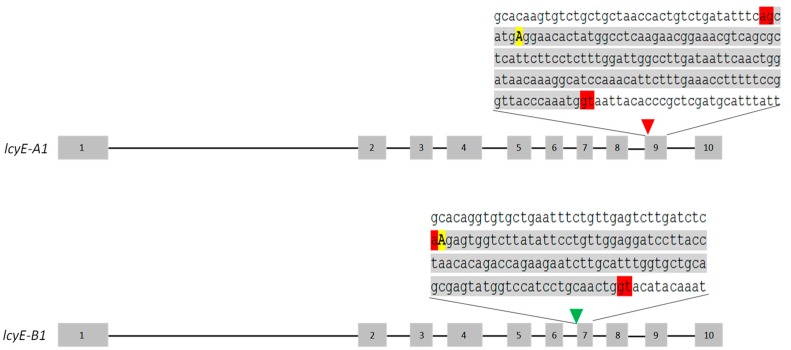
Schematic representation of the two allelic mutations selected for the cross. Grey boxes and black lines indicate exons and introns, respectively. The red triangle indicates the premature stop codon localized on exon 9 of the homeoallele *lcyE-A1* in the mutant line Kronos 2426. The green triangle shows the splice site mutation located in the 3′ region of intron 6 of the *homeoallele lcyE-B1* in the mutant line Kronos 3179. The zooms show the regions containing the mutations, which are highlighted as yellow uppercase letters; the spicing sites are in red.

A preliminary in silico study permitted the identification of two mutant lines possessing deleterious mutations on the alleles *lcyE-A1* and *-B1*, respectively. The line Kronos 2426 had a nonsense mutation in exon 9 of the *lcyE-A1* homeoallele (Figure 2), while the line Kronos 3179 possessed a splice site mutation located in the 3′ region of intron 6 of the homeoallele *lcyE-B1* (Figure 2). The presence of the mutations was confirmed by Sanger sequencing.

To investigate the effect of the splice site mutation, RT-PCR was performed using a primer pair that amplified the region between exons 6 and 7 of *lcyE-B1*. The presence of intron 6 in the *lcyE-B1* transcript was confirmed by sequencing the obtained amplicon. The maintenance of the intron in the mutant allele altered the amino acid sequence starting from amino acid 362. Important and conserved domains are located in the missing region of the mutant LCYE-B1 protein, such as the cyclase domain CM II (fundamental for catalytic activity), the charged region, and the β-LCY region [21,22,23].

### 2.3. Pyramiding the LCYE Null Mutations in the Durum Wheat cv. Kronos

**Figure 3:**
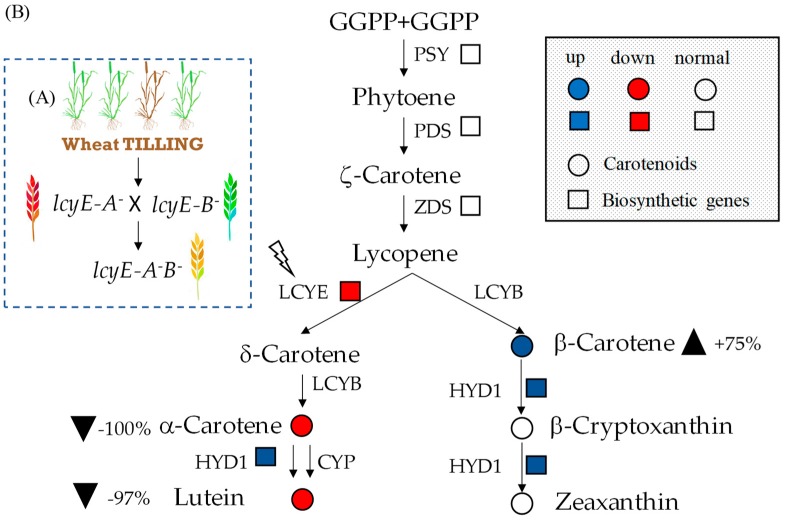
(**A**) Schematic illustration of the strategy used to silence *lcyE* in durum wheat. (**B**) A graphic representation of the changes identified at transcriptional and metabolic level in the seed of the complete null LCYE mutant lines. The squares and circles indicate the biosynthetic genes and carotenoids (metabolites), respectively. The black triangles pointing up indicate an increase of the metabolites, whereas those pointing down indicate a decrease of the metabolites. Blue and red colors indicate an up- or downregulation of gene expression or carotenoid accumulation.

**Figure 4:**
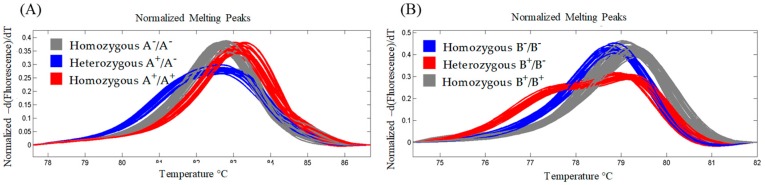
HRM genotyping of a selection of F_2_ progeny bred from the cross LCYE-A1^−^ × LCYE-B1^−^. Derivative melting curves illustrate detection of the heterozygous and homozygous mutant lines and homozygous wild-type plants. For (**A**,**B**), the analyses refer to the homeoalleles *lcyE-A1* and *lcyE-B1*, respectively.

In order to combine the two lcyE null alleles into a single genotype, the F_2_ generation of the cross LCYE-A1^−^ × LCYE-B1^−^ was assayed using HRM genotyping (Figure 3A and Figure 4). The analysis permitted distinguishing the melting curves produced by the amplicons from homozygous, heterozygous, and wild-type genotypes for each homeoallele (Figure 4A,B). Among the 89 F_2_ plants screened, 5 were of genotype LCYE-A1^−^B1^−^, 7 LCYE-A1^−^B1, 3 LCYE-A1B1^−^, and 7 LCYE-A1B1; the remainder were heterozygous for one or both homeologues. The segregation pattern was consistent with digenic inheritance.

### 2.4. Expression Analysis of Major Genes Involved in Carotenoid Biosynthesis

The abundance of *lcyE* transcripts in the complete null LCYE lines was estimated by qRT-PCR. Besides evaluating possible pleiotropic effects due to the presence of deleterious mutations in *lcyE* homeoalleles, the expression of four key genes (β-carotene hydroxylase, HYD1; phytoene desaturase, PDS; phytoene synthase 1, PSY1; and zeta carotene desaturase, ZDS), shown to be involved in the synthesis of carotenoids, was examined.

**Figure 5:**
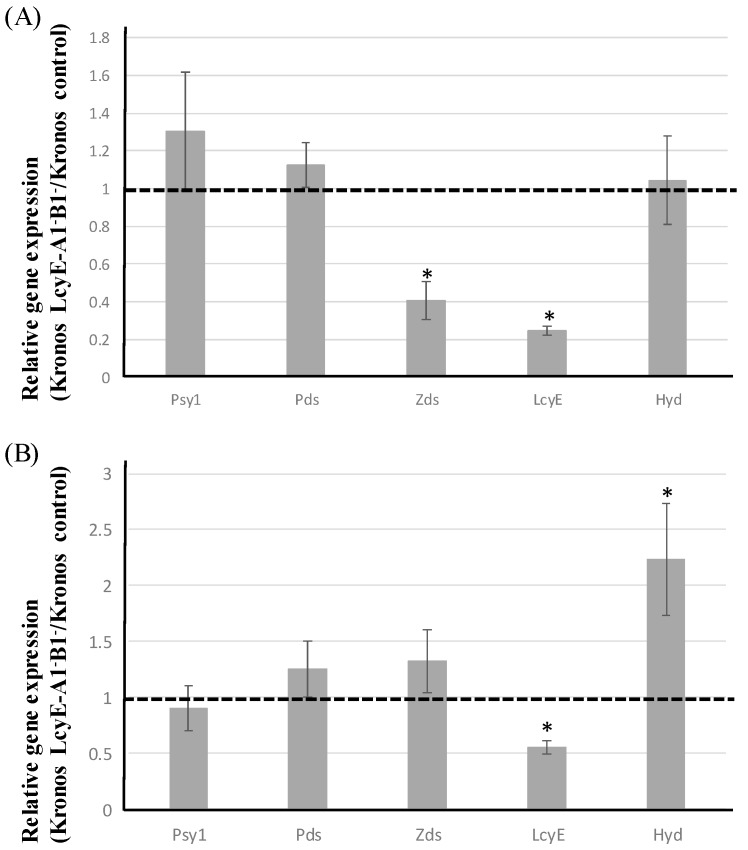
Transcriptional behavior of genes encoding phytoene synthase 1 (PSY1), phytoene desaturase (PDS), zeta carotene desaturase (ZDS), LCYE, and β-carotene hydroxylase (HYD) in leaf (**A**) and grain (**B**). Each bar represents the mean of three biological replicates, each of which was derived from three technical replicates. The data are given in the form of fold differences in transcript abundance between the control and the Targeting Induced Local Lesions in Genomes (TILLING) line Kronos LCYE-A1^−^B1^−^. The dotted line indicates the relative transcription value of the control (cv. Kronos). Standard errors are shown above each bar, along with an asterisk to indicate where the value differed significantly (*p* < 0.05) from that of the wild type.

In leaves, the relative expression of *lcyE* and *zds* was reduced by 80% and 60%, respectively (Figure 5). The expression of the other three key genes did not change between the complete mutant line and the control (Figure 5).

In grains, the abundance of *lcyE* transcript was reduced by more than 40% in the complete null LCYE genotypes compared with the control (cv. Kronos) (Figure 3B and Figure 5). The expression of the gene-encoding β-carotene hydroxylase (*hyd*) was upregulated (by twofold compared with wild type), whereas that of the other genes was unaffected in the mutant lines compared with the control (Figure 3B and Figure 5).

### 2.5. Carotenoids Evaluation in Grain and Leaves

**Figure 6:**
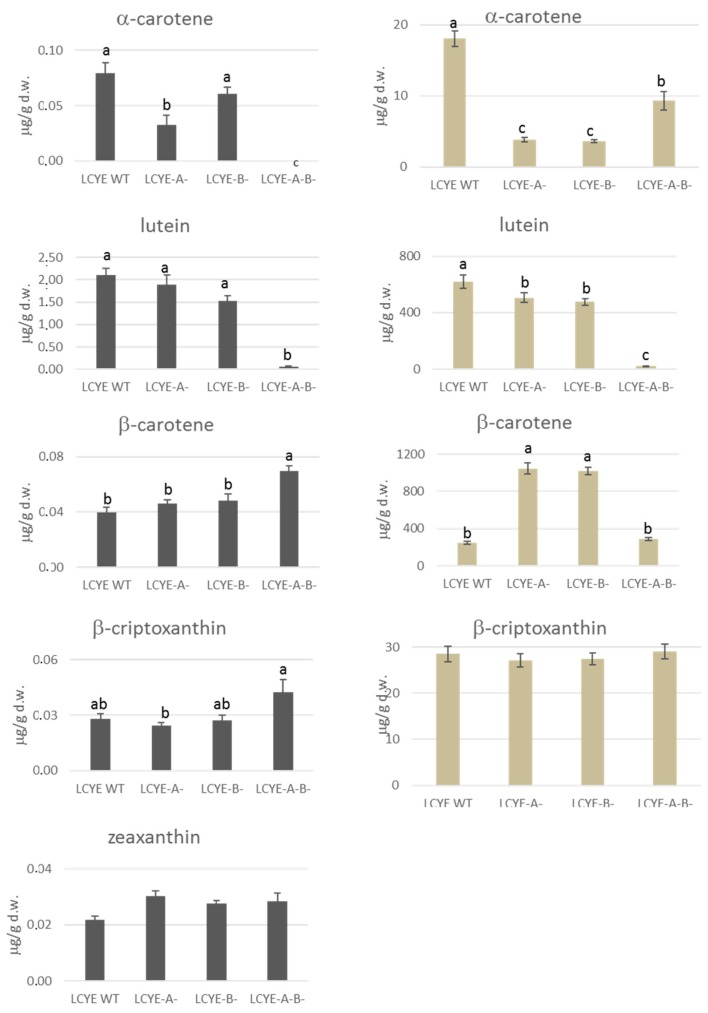
Carotenoid content in grain (black) and leaf (gray) of partial and complete null LCYE mutant lines. Values are reported as micrograms per gram of dried weight. Standard errors are shown above each bar, along with different letters to indicate where the value differed significantly (*p* < 0.05).

To investigate the effect of lcyE mutations on the carotenoid pathway, we performed an analysis for each mutant line of all the metabolites produced from lycopene by the two downstream biosynthetic branches (Figure 6 and Figure S2; Table S1). The analysis of the single carotenoids in the grain confirmed lutein as the most abundant compound in LCYE wild type and even in single mutants LCYE-A^−^ and LCYE-B^−^. On the contrary, the double lcyE null mutant showed a significant reduction in the accumulation of lutein and α-carotene in the grain (−97% and −100%, respectively), indicating a block for this branch of the carotenoid pathway generated by the functional knockout mutations on the *lcyE* homeoalleles (Figure 3B and Figure 6). An opposite trend was observed for the main compounds of the other branch of the pathway that, through the introduction of both β- and ε-rings to lycopene, led to the formation of β-carotene, β-cryptoxanthin, and zeaxanthin. Our results showed a significant increase of 75% in grain β-carotene content compared with the wild type (Figure 3B and Figure 6). Single-locus mutations showed no significant effect on the accumulation of carotenoid compounds. In fact, with the exception of the α-carotene grain content in the LCYE-A^−^ mutant, no other significant differences were detected between the single LCYE-A^−^ and LCYE-B^−^ mutant lines and the wild type.

Possible differences in carotenoid contents were also investigated in LCYE-A^−^, LCYE-B^−^, and LCYE-A^−^B^−^ mutant lines and wild-type leaves (Figure 6). Compared to grains, the amount of carotenoid compounds in the leaves was significantly higher, confirming what was previously reported by Richaud et al. [24]. In the double-mutant lines, the carotenoid profile followed the same pattern as in the grain, displaying the block in the accumulation of lutein (−100%) and a significant reduction in the α-carotene (−50%) compared with the wild type. Unlike grain, in leaves, the quantitative effect produced by the single null lines was greater than that produced by the double mutant, both in the decrease of α-carotene and the increase of β-carotene amounts. Specifically, the content of β-carotene in the double mutant was similar to that of the wild type, while both single-mutant lines recorded an increase of more than 300% compared with the wild type, with no change in β-cryptoxanthin content.

At the phenotypic level, no difference was observed between the mutant lines and the control (cv. Kronos).

## 3. Discussion

Micronutrient malnutrition, and in particular, vitamin A deficiency, remains one of the most serious health problems affecting developing countries. Over the years, individual national governments along with intergovernmental organizations such as the UN (through WHO, FAO, and IFAD) and nongovernmental organizations (NGOs) have carried out numerous campaigns to tackle the problem of malnutrition associated with micronutrient deficiency. Several approaches have been attempted, such as supplementation campaigns and food fortification, but although in some cases they have provided very positive results, they have not been enough to solve this problem definitively. The biofortification of staple crops is an upcoming, promising, sustainable, and long-term strategy to provide to populations affected by micronutrient malnutrition foods improved from a nutritional point of view. In this context, the International Food Policy Research Institute (IFPRI), in collaboration with multiple CGIAR centers and international partner organizations, started the HarvestPlus program, the goal of which is to tackle hidden hunger on a global scale by breeding for vitamins and mineral content for everyday food crops. In particular, HarvestPlus has the target to provide to 1 billion people biofortified crops by 2030.

Since 2000, numerous provitamin A biofortified crops have been released, providing very encouraging results, such as in the case of orange-flashed sweet potatoes [25] and maize [26] used in Africa to counteract vitamin A deficiency. Among the different approaches adopted for biofortification, the transgenic approach certainly provided the best results in terms of micronutrient amount accumulated in the edible parts of plants. β-carotene, the main precursor to vitamin A, has frequently been selected as a target for metabolic engineering interventions aimed at the accumulation of carotenoids in edible plant organs, such as for golden rice [27]. In particular, Ye et al. [27] obtained β-carotene biofortified rice by reconstructing the entire β-carotene biosynthetic pathway into rice endosperm through the introduction of three key genes (phytoene desaturase, ζ-carotene desaturase, and lycopene β-cyclase) that are not present in rice genome. Similarly, Diretto et al. [28] overexpressed a bacterial three-gene minipathway for β-carotene biosynthesis under a tuber-specific promoter in potato, realizing golden potatoes with up to 47 μg/g of β-carotene. A transgenic strategy of metabolic engineering permitted increasing β-carotene by 65-fold (up to 3.21 μg/g) in wheat grain by introducing two bacterial carotenoid biosynthetic genes (*CrtB* and *CrtI*) [29]. Significant levels of β-carotene accumulation (up to 5.06 μg/g with an increase of 31-fold) were obtained in wheat by combining push and block strategies (overexpression of *CrtB* and silencing of *TaHyd*) [15]. The introduction of five carotenogenic genes into a white maize endosperm variety produced transgenic plants containing different carotenoid compositions [30].

Although the transgenic approach is powerful and effective to modulate carotenoid content and composition, GMOs are subject to legal and social limitations that make their cultivation, processing, and marketing difficult or impossible in certain countries. In this context, the biofortification of staple crops, through the TILLING approach, offers numerous advantages, including overcoming the limits imposed by the lack of genetic variability in traditional breeding, the acceleration of breeding programs, and, above all, the possibility of developing new biofortified varieties that do not have the limitations that characterize transgenic organisms [31]. Different bread and durum wheat TILLING platforms have been produced in the last 15 years [32,33,34,35,36,37]. Recently, using the exome capture technique, Krasileva et al. [20] realized a wheat TILLING resource of bread and durum wheat containing 2735 lines. They sequenced the protein coding regions of about 1500 durum [34] and 1200 bread [36] mutagenized lines, identifying about 10 million SNP mutations dispersed on the different chromosomes [38].

In this study, we developed and characterized durum wheat lines in which the genes encoding LCYE enzymes were silenced by EMS treatment based on the TILLING platform of the durum wheat cultivar Kronos [20], with the aim to increase the amount of provitamin A in durum wheat grain. The comparison of amino acid sequences highlighted that the LCYE proteins are strongly conserved among plant species, except for the signal peptide and N-terminal domain, which have great homology only between wheat and barley but differ from other cereals and plant species. The phylogenetic analysis confirmed high relatedness between wheat and barley, which, along with *B. distachyon*, were regrouped in the same cluster. This result agrees with previous investigations, which demonstrated that, despite wheat and barley having diverged about 11 million years ago, the gene structure, order, and content are strongly conserved [39].

Here, the complete silencing of *lcyE* blocked the pathway responsible for the synthesis of α-carotene and lutein and favored the accumulation of β-carotene, which was increased (+75%) in the grain of the durum wheat mutants compared with the control. Using a similar approach and the same TILLING platform [20], Richaud et al. [24] targeted the homeoalleles coding LCYE enzymes. The characterization of the single null mutants did not highlight significant differences between mutants and wild type in wheat grain, suggesting that in polyploid species (such as durum wheat), the pyramiding of single null mutations is necessary to observe a phenotype.

Previous studies demonstrated that the knockout of the *lcyE* gene alters the flux from α-carotene to β-carotene in different plant species, such as maize, potato, sweet potato, and brassica [40,41,42,43]. In maize, the natural genetic variation of *lcyE* was associated with an increased amount of β-carotene (up to 13.6 μg/g) [40]. In polyploid species, such as wheat, the effect of polymorphism on the *lcyE* alleles is masked by the presence of the other homeoalleles; for this reason, it is more complex to identify novel natural allelic variants useful for breeding programs.

Although β-carotene was increased in LCYE complete null lines, the amount of this carotenoid remained rather low in durum wheat grain. A possible explanation is that the upregulation of hydroxylase converted the β-carotene in the other xanthophylls. It is very likely that the simultaneous suppression of *lcyE* and *hyd* could be a successful strategy to obtain wheat genotypes biofortified with higher amounts of β-carotene. Another possibility to explain the low accumulation of carotenoids in wheat is the limited amount of substrate upstream of all-*trans*-lycopene. Regarding this, significant levels of β-carotene accumulation were observed by combining push and block strategies in bread wheat transgenic lines (overexpression of phytoene synthase and silencing of β-ring hydroxylase) [15].

In future works, this study provides a good starting point for further breeding programs aimed at increasing β-carotene content in durum wheat. The combination of the “block strategy” reported here and the natural variability of the carotenoid content present in tetraploid wheat germplasm [44] could contribute to further increasing the concentration of β-carotene in the grain.

In conclusion, to the best of our knowledge, this is the first nontransgenic study of metabolic engineering that modulated the amount of carotenoids in durum wheat grain, enhancing the β-carotene accumulation.

Therefore, considering that global consumption of pasta continues to grow with increasing interest in wellness, this type of study could have a very important impact on the health of a large audience of consumers, not only in developing countries.

## 4. Materials and Methods

### 4.1. Plant Materials

Two LCYE mutants (Kronos 2426 lacking the homeoallele LCYE-A^−^ and Kronos 3179 lacking the homeoallele LCYE-B^−^) were identified through in silico research on the TILLING platform available at University of Davis [20]. These mutants, along with the complete null LCYE-A^−^B^−^ and the control plants (cv. Kronos), were vernalized at 4 °C for 15 days. The growing conditions were 20/24 °C with a 16 h light period and light intensity of 300 μE·m^−2^·s^−1^.

### 4.2. Isolation of LCYE Sequence and Their Phylogenesis

LCYE protein sequences of wheat and different species were isolated from the NCBI database. A phylogenetic analysis was carried out using the neighbor-joining method, as implemented in the MEGA X v10.1 software package (www.megasoftware.net/), applying 1000 bootstrapping replications [45].

### 4.3. High-Resolution Melting Genotyping

F_2_ progeny bred from the cross LCYE-A^−^ × LCYE-B^−^ which lacked functional *lcyE* alleles at both loci were identified using an HRM-based assay. A nested PCR strategy was followed as previously described in Botticella et al. [46]. The second reaction, made up to a volume of 10 μL, included as a template a 1 μL aliquot of a 1:60 dilution of the first reaction, 5 μL GoTaq^®^Hot Start Colorless Master Mix (Promega, Madison, WI, USA), 1 μL LC Green Plus (Idaho Technology Inc., Salt Lake City, UT, USA), and 1.5 μL of each primer to give a final primer concentration of 0.5 μM. The sequence of all of the nested PCR primers used is given in Table S2. The PCR program was described in Sestili et al. [47] and comprised a 95 °C/5 min initial denaturation, followed by 39 cycles of 95 °C/30 s, 60 °C/20 s, and 72 °C/20 s. At the end of the final extension step, the reaction was held at 95 °C for 30 s, then at 25 °C for 60 s. The second PCR was carried out in 96-well Frame-Star plates (4titude Ltd., Surrey, UK) and the Light Scanner instrument (Idaho Technology, Inc.) was used to analyze the melting curves.

### 4.4. Quantitative Real Time-PCR (qRT-PCR)

Total RNA was extracted from leaves and immature grains (15 days postanthesis (DPA)) of greenhouse-grown plants using a Spectrum Plant Total RNA kit (Sigma-Aldrich, St. Louis, MO, USA). A 1 µg aliquot of total RNA was used as a template for the synthesis of ss cDNA, achieved using a QuantiTect Reverse Transcription Kit (Qiagen, Hilden, Germany). qRT-PCR was performed using a CFX 96 Real-Time PCR Detection System device (Bio-Rad, Hercules, CA, USA), following the procedure described by Camerlengo et al. [48]. Each reaction was carried out in a final volume of 15 μL, consisting of 7.5 μL SsoAdvUniver SYBR GRN SMX (Bio-Rad), 0.5 μM of each primer, and 1 μL of cDNA. The protocol for qRT-PCR analyses was 94 °C for 30 s and 40 cycles at 94 °C for 5 s, 60 °C for 30 s, and a melt curve of 65–95 °C with a 0.5 °C increment at 5 s/step. Three β-actin was used as housekeeping gene. Relative gene expressions were calculated using the 2^−ΔΔ*C*t^ method [49]. The primer pairs are listed in Table S2. Each genotype was represented by three biological replicates, each of which, in turn, was associated with three technical replicates.

### 4.5. DNA Sequencing

Genomic DNA was extracted from cv. Kronos and the putative mutant lines using a NucleoSpin^®^Plant II kit (Macherey-Nagel, Düren, Germany). The region containing the mutations were amplified using the primer pairs LCYE-3A (F2 and R2) and LCY-3B (F1 and R2) (Table S2) in 50 μL reactions comprising 100 ng genomic DNA, 1× GoTaq^®^Hot Start Colorless Master Mix (Promega), and 0.5 μM of each primer. The amplification conditions were 95 °C for 3 min, followed from 30 cycles of 95 °C for 1 min, 60 °C for 1 min, and 72 °C for 1 min. A final extension was performed at 72 °C for 5 min. The resulting amplicons were sequenced by Eurofins Genomics (Ebersberg, Germany).

### 4.6. Carotenoid Extraction from Seeds and Leaves

Carotenoid pigment extraction was performed according to Digesù et al. [44] with few modifications. Briefly, the freeze-dried samples of seeds and leaves were milled (Pulverisette 7 Planetary Micro Mill; Classic Line, Fritsch GmbH, Idar-Oberstein, Germany) with an agate jar and balls and stored at −20 °C until analysis. Seeds (0.5 g) and leaves (0.05 g) of each accession were extracted in a screw-capped tube by adding 2 mL of extraction buffer (hexane/acetone, 80:20 *v*/*v*) and 300 μL of butylated hydroxytoluene (BHT) (0.1% *w*/*v*) as an antioxidant and stirred in the dark for 16 h. Samples were then centrifuged for 10 min at 4000 rpm, the supernatants placed in glass tubes, and the residues were extracted once again by adding 2 mL of extraction buffer and stirring in the dark for 2 h. The organic layers were collected and filtered with a gyroscope filter for syringe PTFE (porosity of 0.45 mm). Then, 2 mL of extract was evaporated to dryness under vacuum. Finally, the dry residues were redissolved in 200 μL of MeOH: DCM (45:55 *v*/*v*) for the analysis.

### 4.7. Carotenoid Analysis by HPLC- Diode Array Detector (DAD)

For the carotenoid analysis, a sample volume of 20 μL was injected into an Agilent Technologies 1100 HPLC system equipped with an automatic sampler and a DAD. Separation was done on a YMC C30 column (250 × 4.6 mm i.d., 5 μ). The mobile phase was methanol and methyltert-butyl ether (MeOH: TBME 89:11 *v*/*v*), previously degassed by sonication for 10 min, at a constant flow rate of 1 mL/min. Spectrophotometric detection was achieved in the range of 400–600 nm and peaks were detected at 450 nm. Carotenoids were identified through their characteristic spectra, and comparison of retention times with those of pure standard solutions and their quantification was calculated using the respective calibration curves. Stock solution of each carotenoid standard was dissolved in ethanol, degassed to remove oxygen, and its concentration was determined spectrophotometrically using the Lambert–Beer law [50]. Solutions were diluted in methanol:di-chloromethane (MeOH:DCM 45:55 *v*/*v*) to make calibration curves using six different concentrations of lutein (between 1.44 and 345.84 μg/mL), zeaxanthin (between 5.56 and 111.25 μg/mL), β-cryptoxanthin (between 0.92 and 18.54 μg/mL), α-carotene (between 0.62 and 12.46 μg/mL), and β-carotene (between 0.80 and 31.84 μg/mL). The standards and all the chemicals used were HPLC grade and were from Sigma-Aldrich Chemical Co. (Deisenhofen, Germany).

## Supplementary Materials

Supplementary materials can be found at https://www.mdpi.com/1422-0067/20/22/5703/s1.

## Abbreviations

WHO	World Health Organization
VAD	Vitamin A deficiency
MEP	Methyl-erythrose 4-phosphate
GGPP	Geranylgeranyl pyrophosphate
PSY	Phytoene synthase
PDS	Phytoene desaturase
ZDS	ζ-carotene desaturase
ZISO	ζ-carotene isomerase
CRTISO	Carotene isomerase
LCYB	Lycopene β-cyclase
LCYE	Lycopene ε-cyclase
HYD	β-ring hydroxylases
TILLING	Targeting Induced Local Lesions in Genomes
qRT-PCR	Quantitative real-time PCR
