# Supplementary material for: Provitamin A Biofortification of Durum Wheat through a TILLING Approach
Source: Int J Mol Sci. 2019 Nov 14;20(22):5703. doi: 10.3390/ijms20225703 (PMC6888361; doi:10.3390/ijms20225703)
Supplement: Supplementary file 1 [file ijms-20-05703-s001.pdf]

Supplementary Materials

```
TaLCYE-A1    ME-STGAAISAPFGCRALRWAGQRPLRAAD-----ARRRRVGSGPGP------EQWRSL- 47
TaLCYE-B1    ME-STGAAISAPFGCRALRWAGQRPLRPAD-----ARRRRVGPGPGP------EQWRSL- 47
TaLCYE-D1    ME-STGAAISAPFGCRALRWAGQRPLRPAD-----GRRRRVGPGPGP------EKWRSL- 47
AetLCYE      ME-STGAAISAPFGCRALRWAGQRPLRPAD-----GRRRRVGPGPGP------EKWRSL- 47
HvLCYE       ME-STGAAISAPFGCRALRWAGQRPLRAAD-----ARRRRVSPGPGA------EQWRSL- 47
OsLCYE       MEFSGGATVSAPFGCCRAAWGAAAAGAGAE-GRSRRVVP----RAVE------PRRRGRW 49
PamLCYE      MG-LSGAAISAPLGCRGLPRGAVGGGGKAR-RAEAERWR----WAGA------GRRSGGA 48
BdLCYE       ME-FTGATVSAPFGCRALRGGGQRPLRAAGLAADGRRRRAAGSKPGA------QQWRNNR 53
ZmLCYE       MG-LSGATISAPLGCCVLRCGAVG-GGKAL-KADAERWR----RAGW------SRRVGGP 47
SobLCYE      MG-LSGATISAPLGCRGLRRGAVG-GGKAR-KAEAERWR----RAGW------SRRVGGP 47
PhLCYE       MG-LSGAAISAPLGCRGLPRSAFGGGGKAR-GAEAERWR----RAGA------GRRSGGA 48
AtLCYE       ------------MECVGARNFAAMAV------STFPSWSCRRKFPVVKRYSYRNIRFGLC 42
NtLCYE       ------------MDCIGARNFATMAV------FTCPRFKSLGRRRIMPRKKQP-----IW 37

                                        Plant β-Lcy conserved region
TaLCYE-A1    KASCVATEKP--DEKAAPGL--EFADDEDYVKGGGGELLYVQMQATKAMESQSKIASKLL 103
TaLCYE-B1    KASCVATEKP--DEKAAPGLGVDFADEEDYVKGGGGELLYVQMQATKAMESQSKIASKLL 105
TaLCYE-D1    KASCVATEKP--DEKAAPGLGVEFADEEDYVKGGGGELLYVQMQATKAMESQSKIASKLL 105
AetLCYE      KASCVATEKP--DEKAAPGLGVEFADEEDYVKGGGGELLYVQMQATKAMESQSKIASKLL 105
HvLCYE       KASCVATEKP--DEKAAPGLGVDFADEEDYVKGGGGELLYVQMQATKAMESQSKIASKLL 105
OsLCYE       MVRCVATEKHKDAAARRGGVEVEFADEEDYVKGGGGELLYVQMQASKSMDSQSKISSKLL 109
PamLCYE      KVRCVATEKHDEAA---AAVGVEFADEEDYRKGGGGELLYVQMQATKPMESQSKIASKLL 105
BdLCYE       KVRCVATEKHKDNKAAAAGLGVEFADEEDYVKGGGGELLYVQMQATKAMESQSKIASKLS 113
ZmLCYE       KVRCVATEKHDETAAVGAAVGVDFADEEDYRKGGGGELLYVQMQSTKPMESQSKIASKLS 107
SobLCYE      KVTCVATEKHDETAA----VGVEFADEEDYRKGGGGELLYVQMQATKPMESQSRIASKLL 103
PhLCYE       KVRCVATEKHDETA---AAVGVEFADEEDYRKGGGGEMLYVQMQATKPMESQSKIASKLL 105
AtLCYE       SVRASG--GGSSGSESCVAVREDFADEEDFVKAGGSEILFVQMQQNKDMDEQSKLVDKLP 100
NtLCYE       P-IHMQ--VKCSGNESCVVVKEDFADEEDYIKAGGSELVFVQMQQNKDMDLQSKLSDKLR 94
                          Dinucleotide binding signature
                          V/IXGXGXXGXXXA
TaLCYE-A1    PIA-DETSVLDLVIIGCGPAGLSLAAESANKGLTVGLIGP--DLPFTNNYGVWEDEFKDL 160
TaLCYE-B1    PIA-DETSVLDLVIIGCGPAGLSLAAESAKKGLTVGLIGP--DLPFTNNYGVWEDEFKDL 162
TaLCYE-D1    PIA-DETSVLDLVIIGCGPAGLSLAAESAKKGLTVGLIGP--DLPFTNNYGVWEDEFKDL 162
AetLCYE      PIA-DETSVLDLVIIGCGPAGLSLAAESAKKGLTVGLIGP--DLPFTNNYGVWEDEFKDL 162
HvLCYE       PIA-DETSVLDLVIIGCGPAGLSLAAESAKKGLTVGLIGP--DLPFTNNYGVWEDEFNDL 162
OsLCYE       PIP-DENSVLDLVIIGCGPAGLSLAAESAKKGLNVGLIGP--DLPFTNNYGVWEDEFKDL 166
PamLCYE      PIS-DENSVLDLVIIGCGPAGLSLASESAKKGLTVGLIGP--DLPFTNNYGVWEDEFKDL 162
BdLCYE       PIA-DETSVLDLVVIGCGPAGLSLAAESAKKGLTVGLIGP--DLPFTNNYGVWEDEFKDL 170
ZmLCYE       PIS-DENTVLDLVIIGCGPAGLSLASESAKKGLTVGLIGP--DLPFTNNYGVWEDEFKDL 164
SobLCYE      PIS-DENTVLDLVIIGCGPAGLSLASESAKKGLTVGLIGP--DLPFTNNYGVWEDEFKDL 160
PhLCYE       PIS-DEDSVLDLVIIGCGPAGLSLASESAKKGLTVGLIGP--DLPFTNNYGVWEDEFKDL 162
AtLCYE       PIS-IGDGALDLVVIGCGPAGLALAAESAKLGLKVGLIGP--DLPFTNNYGVWEDEFNDL 157
NtLCYE       QISSAGQTILDLVVIGCGPAGLALAAESAKLGLNVGLVGP--DLPFTNNYGVWEDEFKDL 152

TaLCYE-A1    GLESCIEHVWKDTVVYLDR-NKPIMIGRAYGRVDRDLLHEELLRRCNDAGVTYLNSKVEQ 219
TaLCYE-B1    GLESCIEHVWKDTVVYLDR-NKPIMIGRAYGRVDRDLLHEELLRRCNEAGVTYLNSKVEQ 221
TaLCYE-D1    GLESCIEHVWKDTVVYLDR-NKPIMIGRAYGRVDRDLLHEELLRRCNEAGVTYLNSKVEQ 221
AetLCYE      GLESCIEHVWKDTVVYLDR-NKPIMIGRAYGRVDRDLLHEELLRRCNEAGVTYLNSKVEQ 221
HvLCYE       GLESCIEHVWKDTVVYLDR-NKPIMIGRAYGRVDRDLLHEELLRRCHEVGVTYLNSKVEQ 221
OsLCYE       GLESCIEHVWKDTIVYLDG-NKPIMIGRAYGRVHRDLLHEELLRRCYDAGVTYLSSKVDK 225
PamLCYE      GLESCIEHVWKDTIVYLDN-NKPILIGRSYGRVHRDLLHEELLRRCYEAGVTYLNSKVDK 221
BdLCYE       GLESCIEHVWKDTIVYLDH-NEPIMIGRAYGRVHRDLLHEELLRRCNEAGVTYLNSKVDK 229
ZmLCYE       GLESCIEHVWKDTIVYLDN-NKPILIGRSYGRVHRDLLHEELLKRCYEAGVTYLNSKVDK 223
SobLCYE      GLASCIEHVWKDTIVYLDN-NKPILIGRSYGRVHRDLLHEELLRRCYEAGVTYLNSKVDK 219
PhLCYE       GLESCIEHVWKDTIVYLDN-NKPILIGRSYGRVHRDLLHEELLRRCYEAGVTYLNSKVDK 221
AtLCYE       GLQKCIEHVWRETIVYLDD-DKPITIGRAYGRVSRRLLHEELLRRCVESGVSYLSSKVDS 216
NtLCYE       GLQACIEHVWRDTIVYLDD-ADPILIGRAYGRVSRHLLHEELLKRCVEAGVLYLNSKVDR 211

TaLCYE-A1    IIESPDGHRVVYCERDRKILCRLAIVASGAASGKLLEYEVGGPRVCVQTAYGVEVEVERY 279
TaLCYE-B1    IKESPDGHRVVYCGRGRKILCRLAIVASGAASGKLLEYEVGGPRVCVQTAYGVEVEVERY 281
TaLCYE-D1    IKESPDGHRVVYCGRGHKILCRLAIVASGAASGKLLEYEVGGPRVCVQTAYGVEVEVERY 281
AetLCYE      IKESPDGHRVVYCGRGHKILCRLAIVASGAASGKLLEYEVGGPRVCVQTAYGVEVEVERY 281
HvLCYE       IIESPDGHRVVYCGRDRKILCRLAIVASGAASGKLLEYEVGGPRVCVQTAYGVEVEVERY 281
OsLCYE       IMESPDGHRVVCCEGDREVLCRLAIVASGAASGRLLEYEVGGPRVCVQTAYGVEVEVENN 285
PamLCYE      ITESPDGHRVVCCERGREILCRLAIVASGAASGRLLEYEVGGPPVCVQTAYGVEVEVENN 281
BdLCYE       IIESPDGHRVVYCERGHKILCRLAIVASGAASGKLLEYEVGGPRVCVQTAYGVEVEVEQF 289
ZmLCYE       IIESPDGHRVVCCDKGREIICRLAIVASGAASGRLLEYEVGGPRVCVQTAYGVEVEVENN 283
SobLCYE      IIESPDGHRVVCCDKGREIICRLAIVASGAASGRLLEYEVGGPRVCVQTAYGVEVEVENN 279
PhLCYE       IIESPDGHRVVCCERGREILCRLAIVASGAASGRLLEYEVGGPPVCVQTAYGVEVEVENN 281
AtLCYE       ITEASDGLRLVACDDNNVIPCRLATVASGAASGKLLQYEVGGPRVCVQTAYGVEVEVENS 276
```

```
NtLCYE       IVESTSGHSLVECEGDIVIPCRFVTVASGAASGKFLQYELGGPRVSVQTAYGVEVEVDNN 271
                        CM I                     CM I          CM I
TaLCYE-A1    PYNPSLMVFMDYRDCFKEKFS---HPEEANPTFLYAMAMSSTRVFFEETCLASKDAMPFD 336
TaLCYE-B1    PYDPSLMVFMDYRDCFKEKFT---HPEEANPTFLYAMAMSSTRVFFEETCLASKDAMPFD 338
TaLCYE-D1    PYDPSLMVFMDYRDCFKEKFT---HPEEANPTFLYAMAMSSTRVFFEETCLASKDAMPFD 338
AetLCYE      PYDPSLMVFMDYRDCFKEKFT---HPEEANPTFLYAMAMSSTRVFFEETCLASKDAMPFD 338
HvLCYE       PYDPSLMVFMDYSDCFKEKFS---HPEEANPTFLYAMAMSSTRVFFEETCLASKDAMPFD 338
OsLCYE       PYDPSLMVFMDYRDCFKDKFS---HPEQGNPTFLYAMPMSSTRIFFEETCLASKEAMPFD 342
PamLCYE      PYDPSLMVFMDYRDCFQEKFS---HSEQENPTFLYAMPMSSTRVFFEETCLASKDALPFD 338
BdLCYE       PYDPSLMVFMDYRDCFKENFS---HPEEANPTFLYAMPMSSTRVFFEETCLASKEAVPFE 346
ZmLCYE       PYDPSLMVFMDYRDCFKEEFS---HTEQENPTFLYAMPMSPTRVFFEETCLASKDAMSFD 340
SobLCYE      PYDPSLMVFMDYRDCFKEEFS---HTEQENPTFLYAMPMSSTRVFFEETCLASKDAMSFD 336
PhLCYE       PYDPSLMVFMDYRDCFQEKFS---HSEQENPTFLYAMPMSSTRVFFEETCLASKDAVPFD 338
AtLCYE       PYDPDQMVFMDYRDYTNEKVR---SLEAEYPTFLYAMPMTKSRLFFEETCLASKDVMPFD 333
NtLCYE       PYDPSLMVFMDYRDYVRHDAQ---SLEAKYPTFLYAMPMTKTRVFFEETCLASKDAMPFD 328
                                                           CM II
TaLCYE-A1    LLKKRLMSRLDAMGVRIVKVYEEEWSYIPVGGSLPNTDQKNLAFGAAASMVHPATGYSVV 396
TaLCYE-B1    LLKKRLMSRLDAMGVRIIKVYEEEWSYIPVGGSLPNTDQKNLAFGAAASMVHPATGYSVV 398
TaLCYE-D1    LLKKRLMSRLDAMGVRIIKVYEEEWSYIPVGGSLPNTDQKNLAFGAAASMVHPATGYSVV 398
AetLCYE      LLKKRLMSRLDAMGVRIIKVYEEEWSYIPVGGSLPNTDQKNLAFGAAASMVHPATGYSVV 398
HvLCYE       LLKKRLMSRLDAMGVRILKVYEEEWSYIPVGGSLPNTDQKNLAFGAAASMVHPATGYSVV 398
OsLCYE       LLKKRLMSRLDAMGVHIRKVYEEEWSYIPVGGSLPNTDQKNLAFGAAASMVHPATGYSVV 402
PamLCYE      VLKKRLMYRLDAMGVRILKVHEEEWSYIPVGGSLPNTDQKNLAFGAAASMVHPATGYSVV 398
BdLCYE       LLKKRMMFRLDAMGVRILKVYEEEWSYIPVGGSLPNTDQKNLAFGAAASMVHPATGYSVV 406
ZmLCYE       LLKKRLMYRLNAMGIRILKVYEEEWSYIPVGGSLPNTDQKNLAFGAAASMVHPATGYSVV 400
SobLCYE      LLKKRLMYRLNAMGVRILKIYEEEWSYIPVGGSLPNTDQKNLAFGAAASMVHPATGYSVV 396
PhLCYE       VLKKRLMYRLDAMGVRILKVHEEEWSYIPVGGSLPNTDQKNLAFGAAASMVHPATGYSVV 398
AtLCYE       LLKTKLMLRLDTLGIRILKTYEEEWSYIPVGGSLPNTEQKNLAFGAAASMVHPATGYSVV 393
NtLCYE       LLKKKLMLRLNTLGVRIKQIYEEEWSYIPVGGSLPNTEQKTLAFGAAASMVHPATGYSVV 388
                                                        Charged Region
TaLCYE-A1    RSLSEAPRYASVISDILRNRVYSGQYLPGSSEMSSPSMLAWGTLWPQERKRQRSFFLFGL 456
TaLCYE-B1    RSLSEAPRYASVISDILRNRVYSGQYLPGSSEMSSPSMLAWGTLWPQERKRQRSFFLFGL 458
TaLCYE-D1    RSLSEAPRYASVISDILRNRVYSGQYLPGSSEMSSPSMLAWGTLWPQERKRQRSFFLFGL 458
AetLCYE      RSLSEAPRYASVISDILRNRVYSGQYLPGSSEMSSPSMLAWGTLWPQERKRQRSFFLFGL 458
HvLCYE       RSLSEAPRYASVISDILGNRVYSGQYLPGSSEMSSPSMLAWGTLWPQERKRQRSFFLFGL 458
OsLCYE       RSLSEAPRYASVISDILRNRVYPGEYLPGTSQSSSPSMLAWRTLWPQERKRQRSFFLFGL 462
PamLCYE      RSLSEAPRYASVISDILRNQV-PAQYLPGNSQNYSPSMLGSFDNHRSSQ--QDVARVLGL 455
BdLCYE       RSLSEAPRYASVISDILRNRVYSGQYLPGSSQDSSPSMLAWRTLWPPERKRQRSFFLFGL 466
ZmLCYE       RSLSEAPRYASVISDILGNRV-PAEYMLGNSQNYSPSMLAWRTLWPQERKRQRSFFLFGL 459
SobLCYE      RSLSEAPRYASVISDILNNRV-PAEYLLGNSQNYSPSMLAWRTLWPQERKRQRSFFLFGL 455
PhLCYE       RSLSEAPRYASVISNILRNRV-PAQYLPGNSQNYSPSMLAWRTLWPQERKRQRSFFLFGL 457
AtLCYE       RSLSEAPKYASVIAEILREETTK--QI-----NSNISRQAWDTLWPPERKRQRAFFLFGL 446
NtLCYE       RSLSEAPKCASVLANILRQNHVK--NMLTSSSTTSISTQAWNTLWPQERKRQRSFFLFGL 446

                            β-Lcy motif
TaLCYE-A1    ALIIQLDNKGIQTFFET-FFRLPKWMWQGFLGSTLSSVDLMLFALYMFAIAPNTLRMNLV 515
TaLCYE-B1    ALIIQLDNEGIQTFFES-FFRLPKWMWRGFLGSTLSSADLMLFALYMFAIAPNTLRMNLV 517
TaLCYE-D1    ALIIQLDNEGIQTFFES-FFRLPKWMWRGFLGSTLSSADLMLFALYMFAIAPNTLRMNLV 517
AetLCYE      ALIIQLDNEGIQTFFES-FFRLPKWMWRGFLGSTLSSADLMLFALYMFAIAPNTLRMNLV 517
HvLCYE       ALIIQLDNEGIQTFFET-FFRLPKWMWRGFLGSTLSSVDLMLFALYMFAIAPNNLRMNLV 517
OsLCYE       ALIIQLNNEGIQTFFET-FFRLPKWMWRGFLGSTLSSVDLILFAFYMFTIAPNQMRMNLV 521
PamLCYE      NPFICGSN----TVFILHVCHSSES----------------------IANGPRQT----- 484
BdLCYE       ALIIQLDNEGIQTFFET-FFRLPKWMWRGFLGSTLSSVDLMLFALYMFAIAPNTLRMNLV 525
ZmLCYE       ALIIQLNNEGIQTFFEA-FFRVPRWMWRGFLGSTLSSVDLILFSFYMFAIAPNQLRMNLV 518
SobLCYE      ALIIQLNNEGIQTFFEA-FFRVPKWMWRGFLGSTLSSVDLILFSFYMFAIAPNQLRMNLV 514
PhLCYE       ALIIQLNNEGIQTFFEA-FFRVPKWMWQGFLGSTLSSVDLILFSFYMFAIAPNQLRMNLV 516
AtLCYE       ALIVQFDTEGIRSFFRT-FFRLPKWMWQGFLGSTLTSGDLVLFALYMFVISPNNLRKGLI 505
NtLCYE       ALILQLDIEGIRSFFRA-FFLVPKWMWQGFLGSSLSSADLMLFAFYMFIIAPNDMRKGLI 505

TaLCYE-A1    RHLLSDPTGSAMIRTYLTL--- 534
TaLCYE-B1    RHLLSDPTGSAMIRTYLTL--- 536
TaLCYE-D1    RHLLSDPTGSAMIRTYLTL--- 536
AetLCYE      RHLLSDPTGSAMIRTYLTL--- 536
HvLCYE       RHLLSDPTGSAMIRTYLTL--- 536
OsLCYE       RHLLSDPTGSTMIKTYLTL--- 540
PamLCYE      ----------SPL--------- 487
BdLCYE       RHLLSDPTGSAMIKTYLAL--- 544
ZmLCYE       RHLLSDPTGSSMIKTYLTL--- 537
SobLCYE      RHLLSDPTGSTMIKTYLTL--- 533
PhLCYE       RHLLSDPTGSTMIKTYLTL--- 535
AtLCYE       NHLISDPTGATMIKTYLKV--- 524

NtLCYE       RHLLSDPTGATMIRTYLTF--- 524
```

Figure S1. Polypeptide alignment of LCYE proteins encoded by a selection of plant species. Conserved residues are highlighted in yellow. The characteristic regions of plant LCYE protein are indicated above the sequence: Conserved β-LCY region, Di-nucleotide binding site, Cyclase motifs (CM) I and II, Charged region and β-LCY motif.

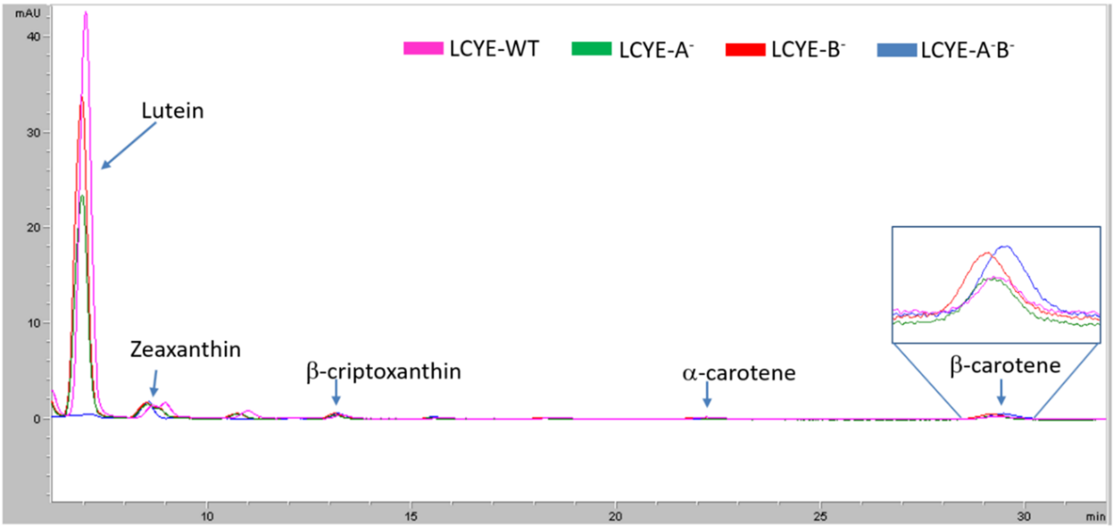

Figure S2. Representative HPLC-DAD chromatograms of grain carotenoids in LCYE-WT (wild type) and, LCYE-A^{-}, LCYE-B^{-}, LCYE-A^{-}B^{-} mutants.

Table S1. Content (µg/g d.w.) of each carotenoid detected in grain and leaf tissues on the biological replicates of WT and mutant accessions. The average values are reported in red. Variation % represent the increment/decrement of mean mutation in comparison to mean WT.

Type Mutation	Accession	Tissue	Lutein	Zeaxanthin	β-Criptoxanthin	α-Carotene	β-Carotene
	LCYE-WT 104-1		2.280	0.020	0.037	0.098	0.041
	LCYE-WT 104-1		2.346	0.020	0.028	0.096	0.033
	LCYE-WT 104-1	grain	2.374	0.020	0.029	0.075	0.030
WT	LCYE-WT 108-2		1.690	0.025	0.021	0.045	0.044
	LCYE-WT 108-2		1.826	0.024	0.025	0.083	0.049
	average		**2.103**	**0.022**	**0.028**	**0.079**	**0.040**
	LCYE-A- E1-2		1.807	0.040	0.020	0.044	0.042
	LCYE-A- E1-2		1.456	0.032	0.036	0.072	0.048
	LCYE-A- E1-2		1.770	0.038	0.022	0.043	0.041
	LCYE-A- C3-2		2.637	0.026	0.025	n.d.	0.058
	LCYE-A- C3-2	grain	2.642	0.024	0.021	n.d.	0.057
A-	LCYE-A- C3-2		2.727	0.028	0.027	n.d.	0.057
	LCYE-A- MS28		1.430	0.029	0.021	0.039	0.038
	LCYE-A- MS28		1.253	0.030	0.021	0.038	0.036
	LCYE-A- MS28		1.352	0.027	0.025	0.056	0.036
	average		**1.897**	**0.030**	**0.024**	**0.032**	**0.046**
	variation %		**-10%**	**+39%**	**-13%**	**-59%**	**+16%**
	LCYE-B- G5-1		1.922	0.028	0.020	0.040	0.049
	LCYE-B- G5-1		1.673	0.028	0.024	0.041	0.045
	LCYE-B- G5-2		1.181	0.026	0.024	0.076	0.030
	LCYE-B- G5-2	grain	1.109	0.023	0.021	0.074	0.033
B-	LCYE-B- 104-4		1.556	0.028	0.029	0.057	0.056
	LCYE-B- 104-4		1.663	0.032	0.033	0.072	0.063
	LCYE-B- 104-4		1.606	0.029	0.040	0.065	0.061
	average		**1.530**	**0.028**	**0.027**	**0.061**	**0.048**
	variation %		**-27%**	**+26%**	**-3%**	**-23%**	**+21%**

A-B-	LCYE-A-B- D2-2	grain	0.027	0.028	0.029	n.d.	0.062
	LCYE-A-B- D2-2		0.028	0.028	0.030	n.d.	0.052
	LCYE-A-B- D2-2		0.031	0.029	0.028	n.d.	0.054
	LCYE-A-B- P2-8		0.050	0.012	0.040	n.d.	0.072
	LCYE-A-B- P2-8		0.072	0.030	0.073	n.d.	0.073
	LCYE-A-B- P2-8		0.089	0.019	0.083	n.d.	0.092
	LCYE-A-B- 103-1		0.099	0.036	0.033	n.d.	0.068
	LCYE-A-B- 103-1		0.060	0.036	0.030	n.d.	0.076
	LCYE-A-B- 103-1		0.064	0.038	0.037	n.d.	0.074
	average		**0.058**	**0.028**	**0.043**	**n.d.**	**0.069**
	variation %		**-97%**	**+30%**	**+52%**	**-100%**	**+75%**
WT	LCYE-WT 104-1	leaf	673.078	n.d.	28.052	18.340	250.678
	LCYE-WT 104-1		766.275	n.d.	33.473	21.322	287.327
	LCYE-WT 104-1		729.165	n.d.	32.440	20.460	275.953
	LCYE-WT 108-2		533.365	n.d.	25.190	16.089	224.013
	LCYE-WT 108-2		518.845	n.d.	23.767	15.170	210.745
	LCYE-WT 108-2		560.340	n.d.	27.720	17.105	229.898
	average		**621.598**	**n.d.**	**28.518**	**18.029**	**245.587**
A-	LCYE-A- E1-2	leaf	605.692	n.d.	31.942	3.608	1218.503
	LCYE-A- E1-2		484.955	n.d.	27.791	3.019	1024.446
	LCYE-A- E1-2		540.358	n.d.	28.926	3.251	1132.812
	LCYE-A- C3-2		450.577	n.d.	24.462	3.361	973.544
	LCYE-A- C3-2		515.624	n.d.	27.331	3.852	1079.698
	LCYE-A- MS28		663.182	n.d.	32.154	5.621	1234.423
	LCYE-A- MS28		356.633	n.d.	19.212	3.177	731.330
	LCYE-A- MS28		433.538	n.d.	24.991	4.356	958.272
	average		**506.320**	**n.d.**	**27.101**	**3.781**	**1044.128**
	variation %		**-19%**	**n.d.**	**-5%**	**-79%**	**+325%**
B-	LCYE-B- G5-1	leaf	445.745	n.d.	24.973	2.731	965.780
	LCYE-B- G5-1		330.002	n.d.	19.773	4.154	758.516
	LCYE-B- G5-1		546.358	n.d.	29.951	3.317	1123.132
	LCYE-B- G5-2		482.682	n.d.	28.223	3.169	1025.669
	LCYE-B- G5-2		472.760	n.d.	26.892	2.981	1005.342
	LCYE-B- 104-4		517.429	n.d.	29.384	4.199	1075.289
	LCYE-B- 104-4		494.082	n.d.	30.785	4.301	1125.585
	LCYE-B- 104-4		530.884	n.d.	29.159	3.974	1049.144
	average		**477.493**	**n.d.**	**27.392**	**3.603**	**1016.057**
	variation %		**-23%**	**n.d.**	**-4%**	**-80%**	**+314%**
A-B-	LCYE-A-B- D2-2	leaf	18.927	n.d.	23.176	17.006	235.177
	LCYE-A-B- D2-2		21.024	n.d.	23.355	12.525	230.319
	LCYE-A-B- D2-2		19.790	n.d.	26.247	11.875	264.986
	LCYE-A-B- P2-8		16.304	n.d.	37.719	10.282	358.305
	LCYE-A-B- P2-8		17.895	n.d.	34.201	8.016	328.246
	LCYE-A-B- P2-8		18.685	n.d.	31.363	6.252	301.383
	LCYE-A-B- 103-1		28.435	n.d.	26.632	6.186	263.228
	LCYE-A-B- 103-1		33.174	n.d.	30.954	6.815	303.999
	LCYE-A-B- 103-1		29.957	n.d.	27.978	4.625	276.341
	average		**22.688**	**n.d.**	**29.069**	**9.287**	**284.665**
	variation %		**-96%**	**n.d.**	**+2%**	**-48%**	**+16%**

Table S2. PCR primer sequences.

Primer name	Sequence (5′-3′)	Target	Genbank acc. N°	Application	Reference
LCY3A F2	AGGAGAAATGAGCACAAGTGTCTGC	ε–lycopene cyclase-A1	EU649785.1	Genotyping/1st PCR	In this paper
LCY3A R2	GGATGTCAAATGTTGCAAC				In this paper
LCY3A-ex9 F	GCTGCTAACCACTGTCTGATA	ε–lycopene cyclase-A1	EU649785.1	Genotyping/2nd PCR	In this paper
LCY3A-ex9 R	GCCAATCCAAAGAGGAAGAATG				In this paper
LCY3B F1	TACAAACGTCATTGTGTACA	ε–lycopene cyclase-B1	EU649786.1	Genotyping/1st PCR	In this paper
LCY3B R2	GGATGCCAAATGTTACAAGG				In this paper
LCY3B-ex7 F	GCTGAATTTCTGTTGAGTCTTGAT	ε–lycopene cyclase-B1	EU649786.1	Genotyping/2nd PCR	In this paper
LCY3B-ex7 R	CTTCTGGTCTGTGTTAGGTAAGG				In this paper
LCYE-B RT Fw	CAAAGAGAAGTTCACACAC	ε–lycopene cyclase	EU649786.1	RT-PCR	In this paper
LCYE 3B-ex7 Rev	CTTCTGGTCTGTGTTAGGTAAGG				In this paper
Actin Fw	CACTGGAATGGTCAAGGCTG	Reference gene	AK450528.1	qRT-PCR	Zhang et al. 2012
Actin Rev	CTCCATGTCATCCCAGTTG				Zhang et al. 2012
ZDS Fw	TTTAGACCTGACCAGAAGACACCA	ζ-carotene desaturase	FJ169496.1	qRT-PCR	Wang et al. 2014
ZDS Rev	AATAACTCCTCTCCAGCACCACA				Wang et al. 2014
PSY Fw	GTTTGGGCCTCTCTGTTGTTG	Phytoene synthase	EF600063.1	qRT-PCR	Wang et al. 2014
PSY Rev	GCCCTCTTGGTGAAGTTGTTG				Wang et al. 2014
PDS Fw	TGAACGCCCCAGTAAACCA	Phytoene desaturase	FJ517553.1	qRT-PCR	Wang et al. 2014
PDS Rev	TTTCCGCCCAACACATCTC				Wang et al. 2014
HDY Fw	ACCACATGGACAAGTTCGAGG	β-ring hydroxylase	JX171671.1	qRT-PCR	Wang et al. 2014
HDY Rev	TTGATCCTGGCGAGCTCCT				Wang et al. 2014
LCYE Fw	ACACACCCTGAGGAAGCCAA	ε–lycopene cyclase	EU649786.1	qRT-PCR	Wang et al. 2014
LCYE Rev	CGCATCCAACCGAGACATCAAC				Wang et al. 2014
